# Profiling study of the major and minor components of kaffir lime oil (*Citrus hystrix DC*.) in the fractional distillation process

**DOI:** 10.11604/pamj.2017.27.282.9679

**Published:** 2017-08-21

**Authors:** Warsito Warsito, Maimunah Hindun Palungan, Edy Priyo Utomo

**Affiliations:** 1 Chemistry Department, Faculty of Natural Science, Brawijaya University, Jawa Timur, Indonesia; 2 Technology of Industry Agryculture Department, Faculty of Agricultur Technology Brawijaya University, Kota Malang, Jawa Timur, Indonesia

**Keywords:** Distillation, kaffir lime oil, pressure, reflux ratio

## Abstract

**Introduction:**

Essential oil is consisting of complex component. It is divided into major and minor component. Therefore, this study aims to examine the distribution of major and minor components on Kaffir lime oil by using fractional distillation. Fractional distillation and distributional analysis of components within fractions have been performed on kaffir lime oil (*Citrus hystrix DC*.).

**Methods:**

Fractional distillation was performed by using PiloDist 104-VTU, column length of 2 m (number of plate 120), the system pressure was set on 5 and 10 mBar, while the reflux ratio varied on 10/10, 20/10 and 60/10, and the chemical composition analysis was done by using GC-MS. Chemical composition of the distillated lime oil consisted of mix-twigs and leaves that composed of 20 compounds, with five main components β-citronellal (46.40%), L-linalool (13.11%), β-citronellol (11.03%), citronelyl acetate (6.76%) and sabinen (5.91%).

**Results:**

The optimum conditions for fractional distillation were obtained at 5 mBar pressure with reflux ratio of 10/10. Components of β -citronellal and L-linalool were distributed in the fraction-1 to fraction 9, hydrocarbon monoterpenes components were distributed only on the fraction-1 to fraction 4, while the oxygenated monoterpenes components dominated the fraction-5 to fraction-9.

**Conclusion:**

The highest level of β-citronellal was 84.86% (fraction-7), L-linalool 20.13% (fraction-5), sabinen 19.83% (fraction-1), and the component level of 4-terpeneol, β-citronellol and sitronelyl acetate respectively 7.16%; 12.27%; 5.22% (fraction-9).

## Introduction

Essential oil is a complex composition that consists of tens to hundreds of compounds. Major components that can be identified in the essential oil include oxygenated monoterpenes, hydrocarbons monoterpene, oxygenated sesquiterpenes, sesquiterpene hydrocarbons, carbonylic compounds, phenols, fatty acids and esters [1]. The chemical composition of essential oil is influenced by several factors, including the level and type of plant species, area, season, temperature, humidity, and other abiotic factors [2, 3]. In general, the composition of essential oil is known as secondary metabolites and classified as terpenoids and polipropenoid [4]. Currently, it has been proven that there are two distinct and independent biosynthetic routes, that is the isopentenyl diphosphate (IPP) route and allylic isomer dimethylallyl diphosphate (DMAPP) route, which produces the two compound groups in plants [5]. These two biosynthetic routes are known as mevalonate pathway to the terpenoids, while phenylpropanoids derived from shikimate pathway [6, 7]. Researchers argue that there is association between the terpenoid formation with enzyme types and enzymatic reaction mechanisms in biosynthetic processes that involves a variety of reactions, such as hydride shift or methyl group, hydration, protonation, cyclization and isomerization. Genetic modification of metabolic pathways provides some promising results to increase the volatile production, therefore, bacteria, yeast and plants are genetically modified for the production of volatile terpenoids derived from shikimic acid. There are several different explanations about the volatile metabolites production by transgenic microorganisms and genetically modified crops [8].

Researchers conclude that this type of approach can be successfully used to generate the level of terpenoids. However, modification of several groups of these compounds is quite difficult because the terpenoids reservoir precursor may not be sufficient for large production of the desired compound [9, 10]. Behrendorff examined the culture media to identify the genes and the production of limonene by using *Saccharomyces cerevisiae*, and use this information to develop a media in limonene biosynthesis process [10]. Terpenoid biosynthesis reaction produces a complex chemical composition of essential oil, both seen from the type of functional group and a carbon skeleton, so almost all kinds of isomer can be found in every type of essential oil. Monoterpenes is the main component of essential oil, whether it is in the form of monoterpenes with open or cyclic carbonic chain. Most of these monoterpenes just have a little difference in the molecular mass and often found as the same isomers. The presence of terpene isomers that have the same structure, physical properties, boiling point, and polarity pushed researchers to develop a method to improve the performance capabilities of GC tool for a quick analysis [11]. Citronellal, geraniol and nerol are the functional isomers of monoterpenoid, while citral is the lemonal from a mixture of terpenoid isomers that is known as the double bond isomers. Citronellal is responsible for the distinctive aroma of lemon, while the geraniol and nerol is the main monoterpenoid alcohol component of rose oil and, palmarosa oil. Isomer is known as geranial or citral A, while the Z-isomer is known as neral or citral B [12]. Z and E configuration difference is caused by the small differences in polarity that affect the strength of interaction between molecules. A number of essential oil components are enantiomers to each other and these compounds only differ in their interaction with polarized light. Enantiomers have identical physical properties, such as boiling point, melting point and spectroscopic spectrum, but it may have different aroma and different taste that can affect the oil quality and also its bioactivity. Results from the enantiomers composition analysis on linalool to 42 types of essential oils by using FSC chiral column with a modified γ-Cyclodextrine (Lipodex E) showed that there are more (-) linalool than (+) limonene, but both are an active antimicrobial, antifungal and antimalarial [13]. The purpose of this study is to examine the distribution of major and minor components on each kaffir lime oil fraction by using fractional distillation, that is performed under reduced pressure and adjusted reflux ratio.

## Methods

*Material and instrument*: The kaffir lime oil sample was obtained from Ngunut, Tulungagung, East Java. Fractional distillation: PiloDist 104-VTU, length = 2 m, and number of stages = 120 (bait 1-2 L). GC-MS spectrometer instrumentation merck Shimadzu QP-type 2010s, RTX-5MS column. *Fractional distillation*: Fractional distillation was performed on lime oil (2 L) by using a PiloDist 104-VTU under reduced pressure, length = 2 m, number of stages = 120, and the conditions were monitored by using a computer. Heating jacket coat were used as a heat source. Fractional distillation was performed repeatedly with varied reflux ratio of 10/10, 20/10 and 60/10, with 0 mbar, 5 mbar and 10 mbar pressure variation. Each fraction is collected and its volume was measured. The fractional distillation was done in Laboratory of Chemistry, LIPI Serpong, Tangerang, West Java.

*Analysis by GC-MS*: Kaffir lime oil and its fractions was analyzed by using a GC-MS Shimadzu type (QP 2010s) with bombardier electron system of 70 eV in voltage, injector temperature was at 280°C, detector temperature at 320°C, pressure foreline: 4 Pa, and the mass range of 30-425 amu). HP-5 (Hewlett Packard-5) capillary column (30 m x 0.25 mm, film thickness 0.25 pM) was used. Gas flow rate on helium carrier was 1 mL/min and the column temperature was programmed as follows: initial temperature 80°C was maintained for 10 minutes, then the temperature was increased from 80°C to 300°C with the increase rate of 4°C/minute. Then the chromatogram was formed.

## Results

**Figure 1:**
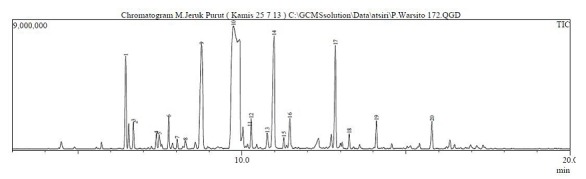
Total ionic chromatogram (TIC) of kaffir lime oil that was obtained from its twig

*The chemical composition of kaffir lime oil*: analysis results on kaffir lime oil from the data obtained by GC-MS total ionic chromatogram (TIC) is shown in Figure 1. It appears that kaffir lime oil is composed of at least 20 compounds, with five main components, as follows: β-citronellal (46.40%), L-linalool (13.11%), β-citronellol (11.03%), citronelyl acetate (6.76%) and sabinene (5.91%). Some components with only > 1% compund are: β-pinene (1.24%), β-micrene (1.27%), tran-β-ocimene (1.56%), (-)-isopulegol (1.57%), 4-terpeneol (1.52%), cis-Linalol oxide (1.86%), trans-β-caryopilene (1.48%) and nerolidol (1.11%), and the other minor compounds.

**Figure 2:**
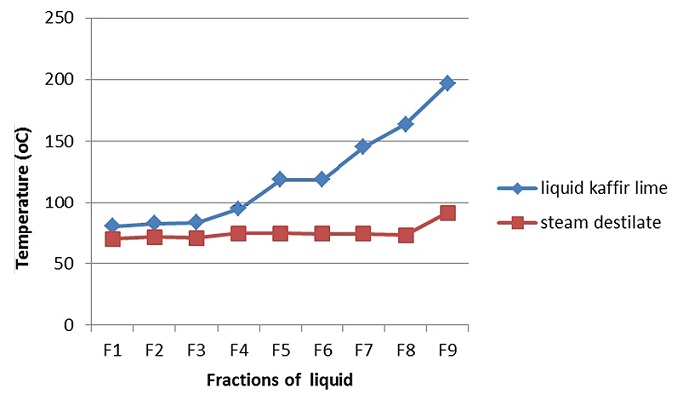
Corelation between the temperature of kaffir lime oil fractions and the stream distillate

**Figure 3:**
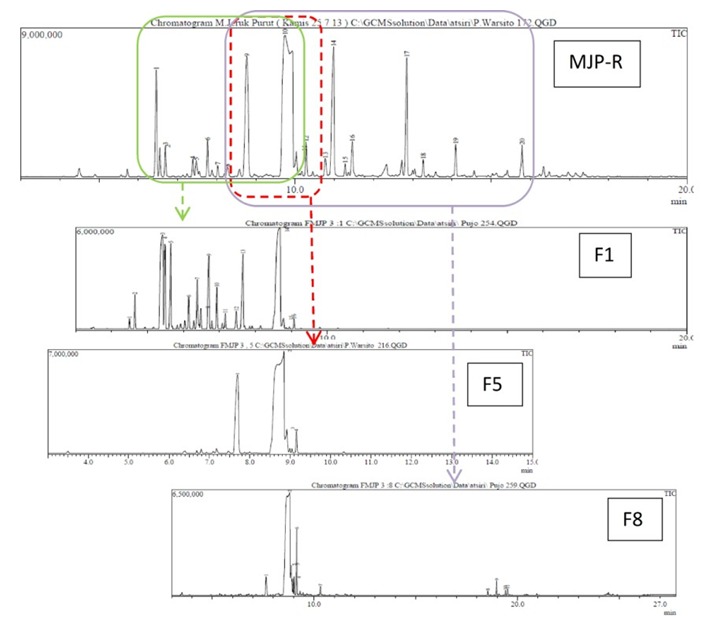
Total ionic chromatogram (TIC); MJP-R: Kaffir lime oil from the twig; F-1; F-5; F-8: Fraction-1, Fraction-5 dan Fraction-8

*Profile of kaffir lime oil in fractination distillation process*: Kaffir lime oil was composed of oxygenated monoterpene and hydrocarbon monoterpene components that only had a small difference in its boiling point. When the kaffir lime oil was heated, the temperature increased gradually from fraction 1 to fraction-9 (Figure 2). The increased fluid temperatures were aligned with the total ionic chromatogram profile. The first fraction (F1) composed of components with a lower molecular mass (boiling point), while the subsequent fractions, such as the F-5 or F-8 was composed of components with a higher boiling point (Figure 3).

**Figure 4:**
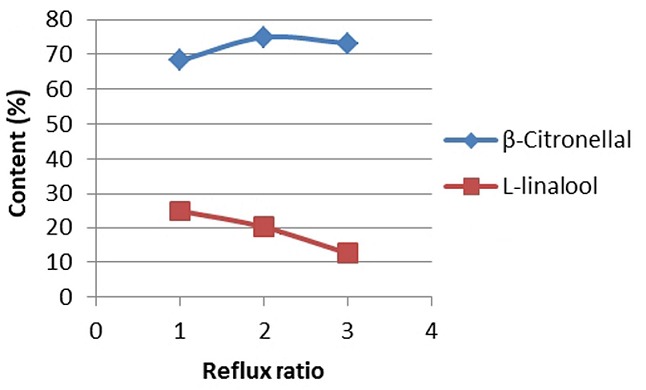
Correlation between reflux ratios (5 mBar pressure) with the level of two main components in fraction-5

*Optimization of reflux ratio and pressures in the fractional distillation of lime oil*: Optimum level of the lime oil main components was generated by the fractional distillation. PiloDist VTU-104 was used as the distillation apparatus, with 120 stages and reflux ratio variation in the reduction of the pressure strength. As the result of the reflux ratio variation, the products partly restored to pass the gray round bottom of the fractionation column, while the other products were collected as distillate. Reflux ratio adjustment on certain fraction was assumed to have capability to produce a significantly increased major component. Early fractions might produce sabinen components at the level of 19.83% (increased by 3 times), β-pinene, β-micren and trans-β-ocimen component, which originally very low in kaffir lime oil, could reach the level of 6.99%, 7.69 % and 7.0% (increased by 5-fold) (Figure 4).

**Figure 5:**
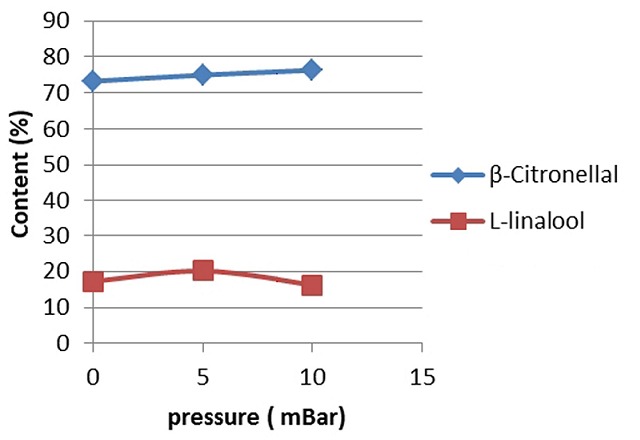
The level of two main components of fraction 5 and several variations of pressure

**Figure 6:**
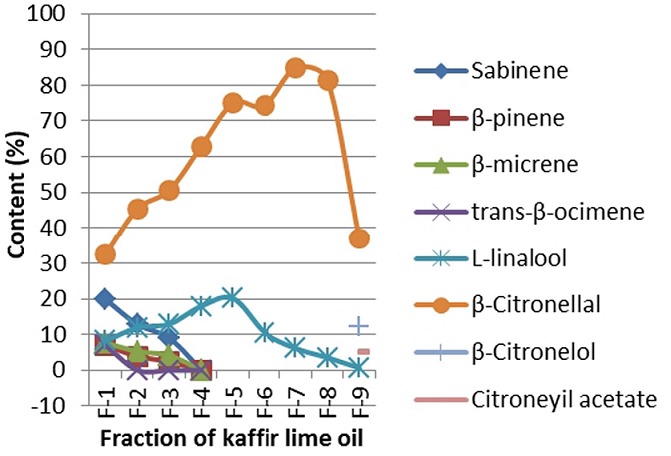
Distribution profile of the main components from the fractional distillation on optimum conditions (reflux ratio of 20/10, 5 mbar pressure)

In this research, the determination of optimum pressure for each treatment of fractional distillation system by using PiloDist 104 VTU 120 stages was based on the distribution pattern of the two components level, ß-citronellal and L-linalool. Figure 5 showed a graph for the fractional distillation process. Application of optimum conditions in the fractional distillation process, i.e. reflux ratio 20/10 and 5 mbar pressure, was performed to separate the distribution pattern of the two major components, as shown in Figure 6.

## Discussion

TIC results showed that the five major components of the kaffir lime oil separated very well with the RTX-5 MS column, with the order of retention time, tR, retention time ie by silica gel column filler increases. TR sequences of these compounds were sabinene, L-linalool, β-citronellal, citronellol and β-sitronelyl acetate. Apart from sabinene, the four other major components contained oxygen, so it was possible to form hydrogen bonds that could result in higher boiling point. This sequence illustrated the boiling point of the compounds. The β-citronellal level that was obtained from the kaffir lime plant twigs (46.40%) was lower than the ß-citronellal level that was obtained from lime plant leaves, which reached 70.30% to 80.04% (analysis product of PT. Indesso). It means that the β-citronellal secondary metabolites stored more in leaves than in twigs. Although the quantitative component is varied, but in general the main component of lime oil is relatively the same with kaffir lime oil from other countries, such as from Johor and Selangor, Malaysia by Loh [14], from Thailand by Srisukh, et al [15]. Distilled kaffir lime oil fractionation at the optimum conditions showed several things, there are: (1) the highest level of β-citronellal in fraction-7 was at 84.86% (increased by 38.46%). He distilled fraction in kaffir lime oil has higher level of β-citronellal and is relatively high compared to β-citronellal that was obtained from steam distillation of kaffir lime leaves (80.04%) [15]. Furthermore, L-linalool components in fraction-5 reached 20.13% (increased by 7.02%), (2) β-citronellal and L-linalool components was distributed in every fraction, which means that during the distillation process, the second component composed the majority of the steam product, (3) sabinen in fraction-1 reached the highest level of 19.83% (increased by 13,92%), it indicates that sabinen has the lowest boiling point among the major components on kaffir lime oil, (4) β-pinene, β-micren and trans-β-ocimen was very low, these components appeared to have significant level value in fraction-1 to fractions-3, although these component became weaker after fraction-4,and (5) 4-terpeneol, β-citronellol and citronelyl acetate appeared on the final fraction, i.e. the fraction-9, respectively in level of 7.16% (increased by 5.64%), 12.27% (increased by 1.24%) and 5.22%, which indicates that all of these compounds has the highest boiling point. It is due to the two components that contains the highest amount of oxygen and are able to form hydrogen bonds.

## Conclusion

This study can be conclude that the highest level of β-citronellal was 84.86% (fraction-7), L-linalool 20.13% (fraction-5), sabinen 19.83% (fraction-1), and the component level of 4-terpeneol, β-citronellol and sitronelyl acetate respectively 7.16%; 12.27%; 5.22% (fraction-9).

### What is known about this topic

- Essential oil is consisting of complex component which is divided into major and minor component.

### What this study adds

- This study showed that the optimum conditions for fractional distillation were obtained at 5 mBar pressure with reflux ratio of 10/10;
- This study give information that component with highest level are β-citronellal with 84.86%.

## Competing interests

The authors declare no competing interest.
